# ^1^H-NMR metabolomics reveals a multitarget action of *Crithmum maritimum* ethyl acetate extract in inhibiting hepatocellular carcinoma cell growth

**DOI:** 10.1038/s41598-020-78867-1

**Published:** 2021-01-13

**Authors:** Davide Gnocchi, Laura Del Coco, Chiara Roberta Girelli, Francesca Castellaneta, Gianluigi Cesari, Carlo Sabbà, Francesco Paolo Fanizzi, Antonio Mazzocca

**Affiliations:** 1 Interdisciplinary Department of Medicine, University of Bari School of Medicine, Piazza G. Cesare, 11, 70124 Bari, Italy; 2 Department of Biological and Environmental Sciences and Technologies, University of Salento, 73100 Lecce, Italy; 3 Department of Organic Agriculture, CIHEAM—Mediterranean Agronomic Institute of Bari, 70010 Valenzano, BA Italy

**Keywords:** Hepatocellular carcinoma, Biochemical networks, Preclinical research, NMR spectroscopy

## Abstract

Hepatocellular carcinoma (HCC) is nowadays the sixth cause of tumour-related deceases worldwide, estimated to become the third in Western countries by 2030. New drugs for HCC treatment still have many adverse effects. Several lines of evidence indicate that plant metabolites offer concrete opportunities for developing new therapeutic strategies for many diseases, including cancer. We previously reported that ethyl acetate extract of a spontaneous edible plant harvested in Apulia, *Crithmum maritimum*, significantly inhibited cell growth in HCC cells. By ^1^H-NMR spectroscopy, here we show that *Crithmum maritimum* ethyl acetate extract counteracts the Warburg effect, by reducing intracellular lactate, inhibits protein anabolism, by decreasing amino acid level, and affects membrane biosynthesis by lowering choline and phosphocholine. Also, we observed an effect on lipid homeostasis, with a reduction in triglycerides, cholesterol, monounsaturated fatty acids (MUFA), and diunsaturated fatty acids (DUFA), and an increase in polyunsaturated fatty acids (PUFA). Taken together, these data demonstrate that *Crithmum maritimum*-induced cytostasis is exerted through a multi-effect action, targeting key metabolic processes in HCC cells. Overall, our findings highlight the role of *Crithmum maritimum* as a promising tool for the prevention and the improvement of the therapeutic options for HCC and other types of tumours.

## Introduction

Hepatocellular carcinoma (HCC) is currently the sixth principal cause of tumour-related deceases worldwide and it is expected to grow into the third one in the Western world by 2030, regardless of the reduced incidence of chronic hepatitis infections^1^. This trend finds the basis in the increased prevalence of metabolic diseases, such as metabolic syndrome (MetS), diabetes, obesity, and non-alcoholic fatty liver disease (NAFLD)^2^. The therapy of HCC is mainly based upon surgery and pharmacology employing tyrosine-kinase inhibitors, such as sorafenib, regorafenib, and levantinib^2^, often in combination with immunotherapeutic agents, for instance, pembrolizumab and nivolumab^3^. Such pharmacological approaches result in many unfavourable consequences and are not tolerated by patients in the long range^4,5^. Thus, innovative and effective therapeutic strategies, with fewer side effects, are extremely needed for HCC.

Plant-derived products and extracts are gaining consideration for therapeutic purposes, and noteworthy effects have been described concerning many pathologies^6,7^, including cancer^8^. We previously demonstrated that ethyl acetate extract of a spontaneous edible plant harvested in Apulia, *Crithmum maritimum* L., exerted a significant inhibitory effect on cell growth in HCC cells by affecting cell cycle regulation and apoptosis^9^. Metabolomics offers a trustable and effective tool to investigate the behaviour of biological systems by analysing their metabolic fingerprint^10^. Many applications have been described so far in the characterisation of the metabolic profile of plants^11,12^, as well as in the assessment of changes of the metabolic signature after a selected treatment, such as plant-derived medicines and conventional drugs^13^, or plant-derived metabolites^14^. Here, we aimed at further characterising the effect of *Crithmum maritimum* in HCC cells by using a metabolomic approach. We, therefore, characterised the effect of the ethyl acetate extract of *Crithmum maritimum* on the metabolic profile in two HCC cell lines, Huh7 and HepG2, by analysing, with ^1^NMR-spectroscopy, cell pellets (aqueous and lipid fractions) and cell culture media. Moreover, we analysed the whole plant powder and the four extracts to determine their metabolite composition. Our results show that ethyl acetate extract of *Crithmum maritimum* substantially changes the metabolic profile in both HCC cell lines. In particular, in the aqueous fraction, we observed a decrease in intracellular lactate, many amino acids, choline, and phosphocholine. Interestingly, the decrease of intracellular lactate in the aqueous fraction was paralleled by the increase in cell culture media, where an increase of glucose was also measured. Notably, we also found that ethyl acetate extract of *Crithmum maritimum* modulates lipid homeostasis, by decreasing triglycerides, cholesterol, and monounsaturated fatty acids (MUFA) and di-unsaturated fatty acids (DUFA), and increasing polyunsaturated fatty acids (PUFA). Moreover, the metabolite composition of the whole plant powder and the ethyl acetate extract offers interesting insights for a better understanding of the broad effect shown by *Crithmum maritimum*. Overall, our findings show for the first time the multitarget action of *Crithmum maritimum* extract in inhibiting HCC cell growth as well as the reversion of the pathologic metabolic profile of HCC cells towards normal patterns.

## Results

### Preparation of *Crithmum maritimum* extracts and their effect on cell proliferation in HCC cell lines

**Figure 1:**
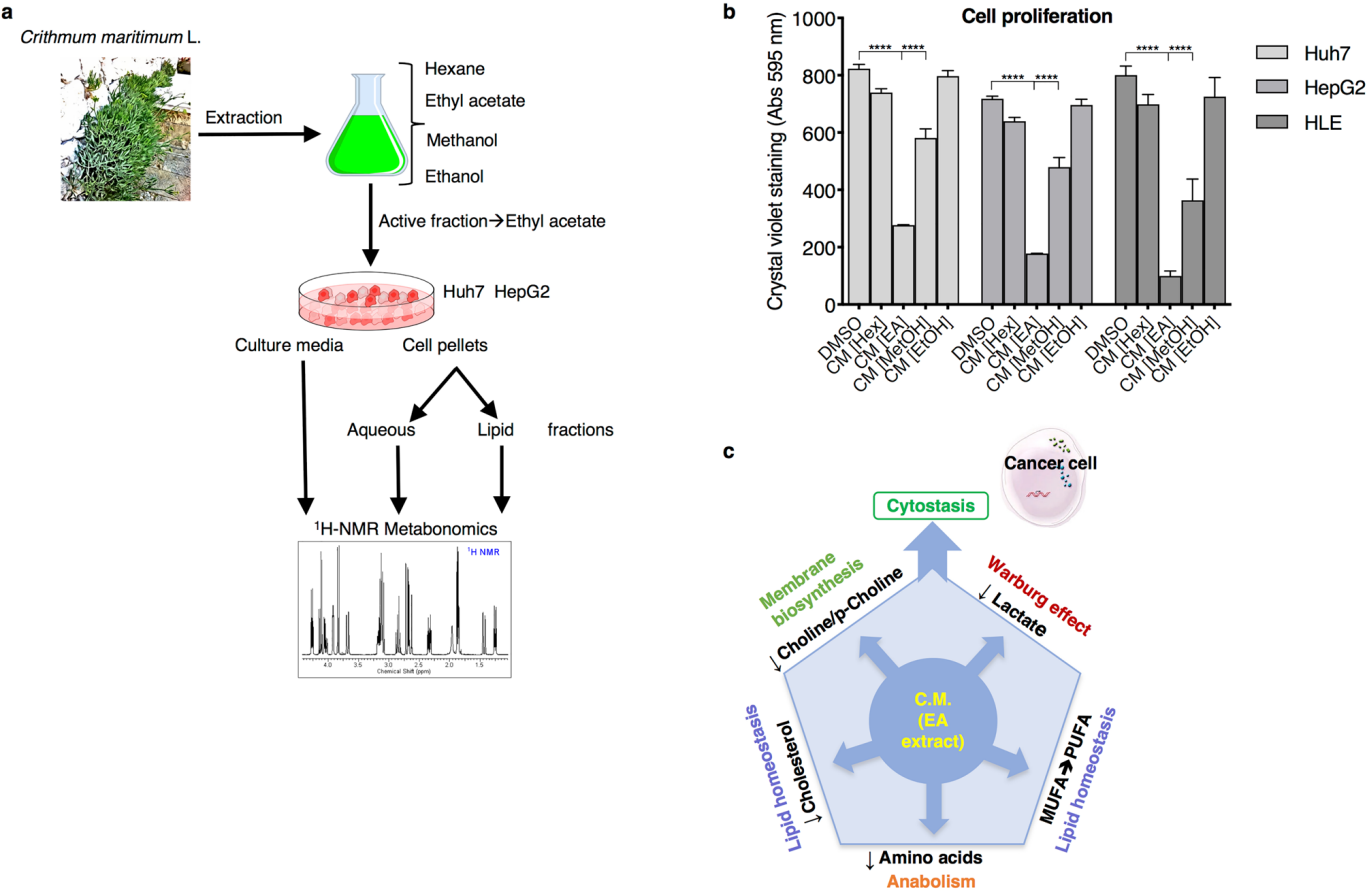
(**a**) Cartoon outline of the experimental workflow (**b**) Effect of the different *Crithmum maritimum* extracts on cell proliferation in three different HCC cell lines (Huh7, HepG2, HLE) as determined by crystal violet staining. Cells were treated for 72 h with the four different *Crithmum maritimum* extracts at 0.5 μM. **** p < 0.0001, *** p < 0.001 as determined by 2-way ANOVA analysis followed by Dunnett’s multiple comparisons tests. (**c**) Cartoon showing the proposed multi-target effect exerted by *Crithmum maritimum* ethyl acetate extract on HCC cells. *DMSO* Vehicle treatment, *CM* *Crithmum maritimum* treatment, *[Hex]* Hexane, *[EA]* Ethyl Acetate, *[MetOH]* Methanol, *[EtOH]* Ethanol. Results are expressed as the mean ± s.e.m. of at least three independent biological replicates, each conducted in triplicate.

As a first step, we prepared extracts from *Crithmum maritimum* lyophilized powder, as described in the Materials and Methods section. The workflow from plant lyophilized powder preparation to ^1^H-NMR analysis is summarised in Fig. 1a. We preliminary analysed the effect of the four different extracts (hexane, ethyl acetate, methanol and ethanol) on cell proliferation in three different HCC cell lines (HepG2, Huh7, HLE) by treating the cells for 72 h with 0.5 μM extract concentration. Results show that the ethyl acetate extract was the most effective in inhibiting cell growth (Fig. 1b). Thus, we focused on this extracts for the following ^1^H-NMR experiments on cell extracts and cell culture media. To corroborate the effect of *Crithmum maritimum* in other tumour histotypes, we carried out the same experiment in a non-HCC cell line, namely HeLa, and observed a similar antiproliferative effect (see Supplementary Fig. S1 online). Also, since it has been shown that the extracellular acidic pH characterising most cancers can hamper the effectiveness of many anti-tumour drug^15,16^, we verified the effect of the four different extracts on cell proliferation under acidic pH conditions. We found that the effect of *Crithmum maritimum* was preserved under these conditions, although HepG2 cells displayed a slight resistance compared with Huh7 (see Supplementary Fig. S2 online).

The combined multi-target action exerted by *Crithmum maritimum* ethyl acetate extracts resulting in the cytostatic effect on HCC cells is illustrated in Fig. 1c.

### ^1^H-NMR characterisation of ***Crithmum maritimum*** ethyl acetate extract

**Figure 2:**
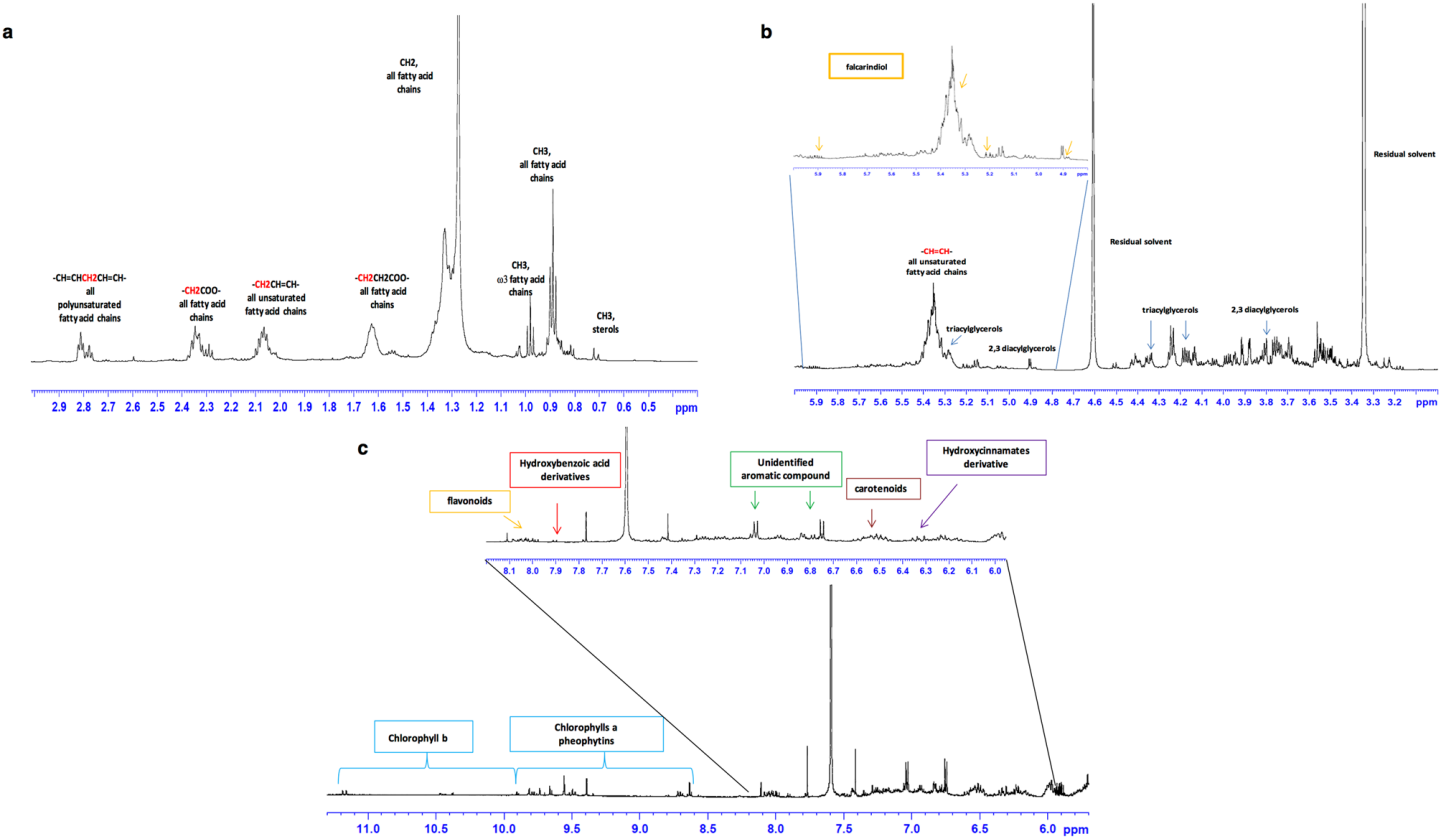
^1^H-NMR typical spectrum (600 MHz, CD_3_OD:CDCl_3_) of *Crithmum maritimum* ethyl acetate extract sample in (**a**) high, (**b**) middle, and (**c**) low-frequency regions, showing the main metabolite classes associated with principal peaks.

Then, we characterised by ^1^H-NMR *Crithmum maritimum* ethyl acetate extract. Visual inspection of the ^1^H-NMR spectrum revealed the presence of different classes of metabolites (Fig. 2). These include fatty acids, polyacetylenes, and in the aromatic overcrowded region, phenolic compounds, and photosynthetic pigments (Fig. 2). Characteristic signals of sterols, tri- and diacylglycerols, and fatty acids were identified (Fig. 2a). The presence of sterols was assessed based on diagnostic resonances of CH_3_-groups at 0.7 and 0.71 ppm, clearly differentiated from the other signals. In particular, signals at 0.71 (C*H*_*3*_-18) and 1.01 (C*H*_*3*_-25) were assigned to β-sitosterol, as also confirmed by the carbons at 12.2 ppm and 19.1 ppm, respectively. The ^1^H resonance at 0.70 ppm was identified as stigmasterol^17^, while the smaller signal observed at 0.55 ppm was assigned to a sterol molecule. *Crithmum maritimum* and halophytes in general are known to be important sources of phytosterols^18^.

Terminal methyl groups (C*H*_*3*_) of all fatty acid chains were observed at 0.88 ppm. A more de-shielded signal at 0.98 ppm was assigned to ω3-fatty acids methyl groups. Broad signals in the range 1.26–1.39, 1.59–1.67, and 2.25–2.41 ppm were detected and identified as characteristic for methylene (n-C*H*_*2*_), β-methylene, and α-methylene (-C*H*_*2*_COO-) protons adjacent to the carbonyl group of all fatty acids. The unsaturated fatty acids were identified by the diagnostic resonances of allylic (–C*H*_*2*_CH=CH–) and olefinic (–C*H*=C*H*–) protons at 2–2.10 and 5.29–5.45 ppm respectively. The presence of polyunsaturated fatty acids such as linoleic (C18:2 ω6) and linolenic (C18:3 ω3) was assessed by the presence of bis-allylic protons (–CH=CHC*H*_*2*_CH=CH–) at 2.74–2.82 ppm. Unsaturated fatty acids are highly represented in wild plants and a significant amount of ω3 and ω6 series was previously reported in *Crithmum maritimum* leaves^19,20^. Several signals characterized the region between 3.5 and 4.6 ppm (Fig. 2b). Among them, the resonances at 4.17 and 4.35 ppm were assigned to glycerol 1–3 protons of triacylglycerols. Partially overlapped with olefinic (–C*H*=C*H*–) protons was identified at 5.28 ppm. A doublet at 4.90 ppm (*J* 3.78 Hz) coupled with a proton signal at 3.80 and a ^13^C at 99 ppm in the HSQC spectrum was assigned to the anomeric proton typical of glycodiacylglycerols sugar moiety. Digalactosyldiacylglycerol is known to be the major glycerolipids of plastids in plants^17^.

Based on 2D experiments and by comparison with previously reported data^21,22^, diagnostic resonances at 5.91 C*H*(2), 5.22 C*H*(1), and 4.90 C*H*(3), together with the spin system at 0.88 (–*CH*_*3*_), 1.26–1.39 (n-C*H*_2_–), 5.29–5.45 (–C*H*=C*H*–) were assigned to falcarindiol. Interestingly, these resonances were not observed in the ^1^H-NMR spectra of the other three fractions (Supplementary Fig. S4-S6 online). Polyacetylene molecules are widely distributed within the Apiaceae family and show many biological and bio-functional activities, such as antibacterial, cytotoxic, and antimutagenic^21,23^.

In the aromatic region (Fig. 2c), a complex pattern of resonances, with several weak signals, in the range 6.4–6.6 ppm was assigned to the conjugated double bonds of carotenoids^17,22^. Despite a lower content for the other Apiaceae species, *Crithmum maritimum* is considered a good source of carotenoids, known for their important antioxidant properties^20^. Characteristic signals for the vinyl protons at 6.32 ppm (J = 15 Hz) and 7.60 ppm (J = 15 Hz) are compatible with caffeoyl moieties of hydroxycinnamic acids, such as chlorogenic acid, reported to be major phenolic compounds in *Crithmum maritimum* aerial parts^20,24,25^. Interestingly, strongly coupled doublets at 7.0 and 6.78 ppm were not observed in the methanol extract (see Supplementary Fig. S5 online). Hydroxybenzoic acid derivatives, whose presence was already reported in the phenolic profile of *Crithmum maritimum* infusions and decoctions^25^, were also identified based on coupled signals at 7.90 and 6.85 ppm. The *J-resolved* spectrum showed a singlet at 6.61 ppm that identified epigallocatechin H2 and H6 aromatic protons. This flavonol was reported to be one of the main flavonoids found in *Crithmum maritimum* leaves^20^. According to the literature, other flavonoids derivatives, characterized by signals at 8.08 and 6.30, were ascribed to rutin, apigenin, catechin, epicatechin, and quercetin^20,25^. Signals from chlorophylls and their metal-free derivatives, such as pheophytins, were identified at the downfield region of the spectrum, between 11.2–8.5 ppm^26^. Observed peaks were attributed to the tetrapyrrole ring N–H protons signals^17^. In particular, signals at 11.16, 11.18, 10.47, 10.37, and in the range 9.95–9.6 ppm were assigned to chlorophyll b, while chlorophyll a and its pheophytins derivatives resonances were observed in the range 9.6–9.32 ppm and 8.53–8.1 ppm^26,27^.

**Figure 3:**
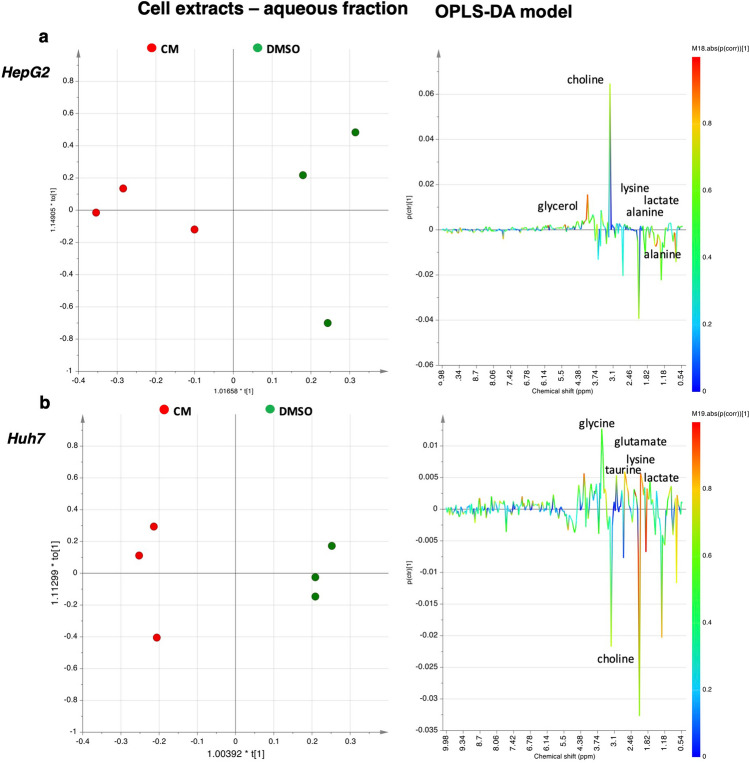
OPLS-DA scores plots (left panel) and corresponding coefficient loading plots (right panel) derived from the ^1^H-NMR spectra of HepG2 (**a**) and Huh7 (**b**) cell extracts aqueous fraction obtained from different groups. *DMSO* Vehicle treatment, *CM* *Crithmum maritimum* treatment.

**Figure 4:**
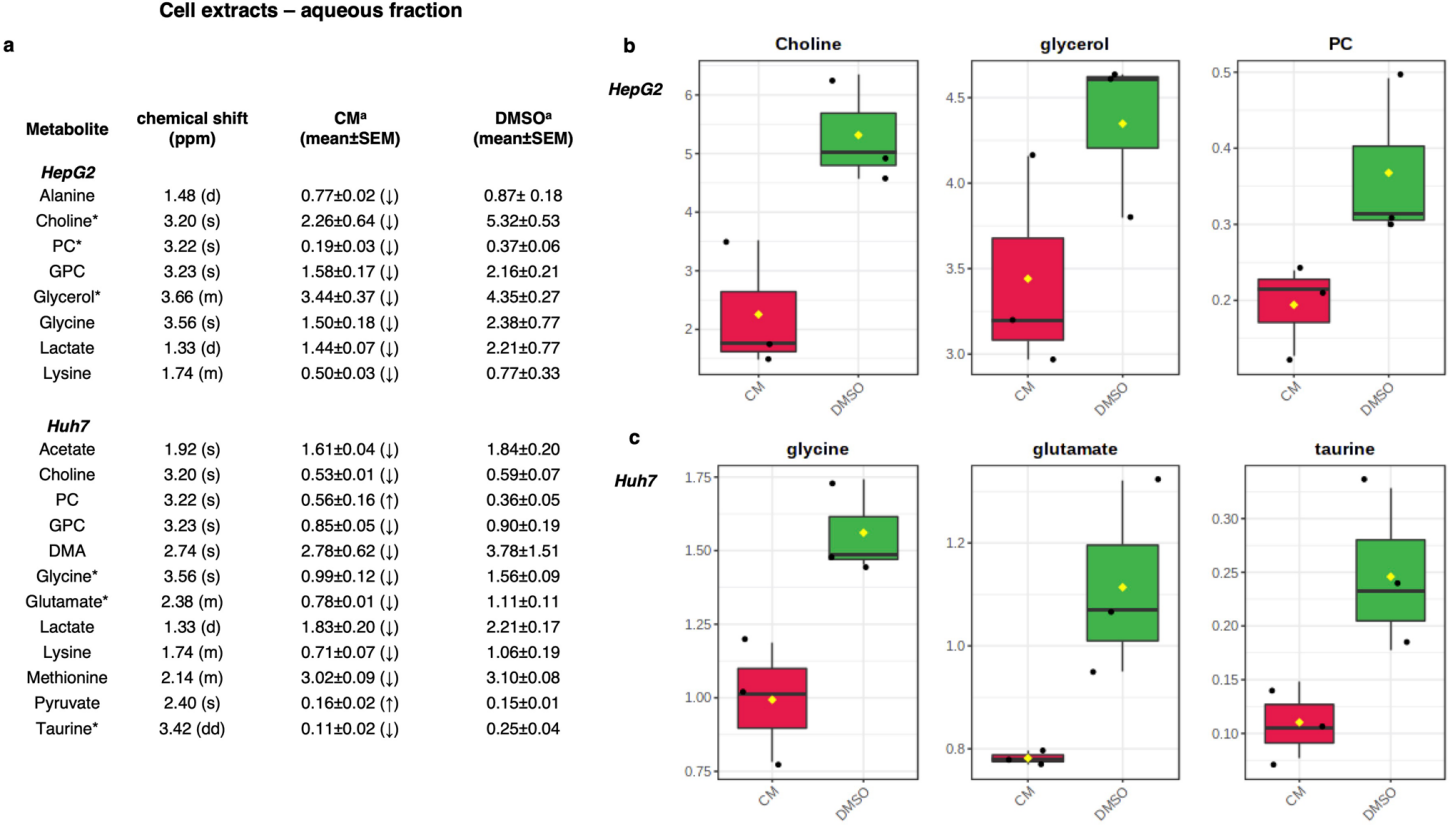
(**a**) Quantitative comparison of metabolites found in the aqueous cell extract. Mean and relative standard error mean refers to the relative integrals of metabolites, determined from cell extract 1D ^1^H-NMR spectra of each group (CM and DMSO treatments). Letters in parentheses indicate peak multiplicities (s, singlet; d, doublet, dd, doublet of doublet; m, multiplet). *DMA* dimethylamine, *PC* *O*-phosho-choline, *GPC* *sn*-glycero-3-phoshocholine. *A *p*-value threshold of 0.05 was obtained from t-test analysis. (**b**,**c**) Box-and-Whisker plots illustrating the trend of significant metabolites grouped according to CM: *Crithmum maritimum* treatment; DMSO: Vehicle treatment**,** derived from the ^1^H NMR spectra of (**b**) HepG2 and (**c**) Huh7 aqueous cell extracts. *DMSO* Vehicle treatment, *CM* *Crithmum maritimum* treatment.

By using ^1^H-NMR, we also analysed the metabolite profile of *Crithmum maritimum* powder and the other three fractions obtained by hexane, methanol, and ethanol extraction. Results are shown in Supplementary Figures S3-S6 online (Figs. 3 and 4).

### ***Crithmum maritimum*** ethyl acetate extract modifies metabolite profile in aqueous cell extracts as evaluated by ^1^H-NMR spectroscopy

We next evaluated by ^1^H-NMR spectroscopy the effect of *Crithmum maritimum* ethyl acetate extract on metabolite profile in two HCC cell lines (Huh7 and HepG2). Cells were treated for 48 h with the extract at the concentration of 0.5 μM, which was used in our previous work. Cell culture media were collected for further analyses. Then, cells were harvested and the pellet obtained after centrifugation was used for the analysis performed on both aqueous and lipid fractions.

**Figure 5:**
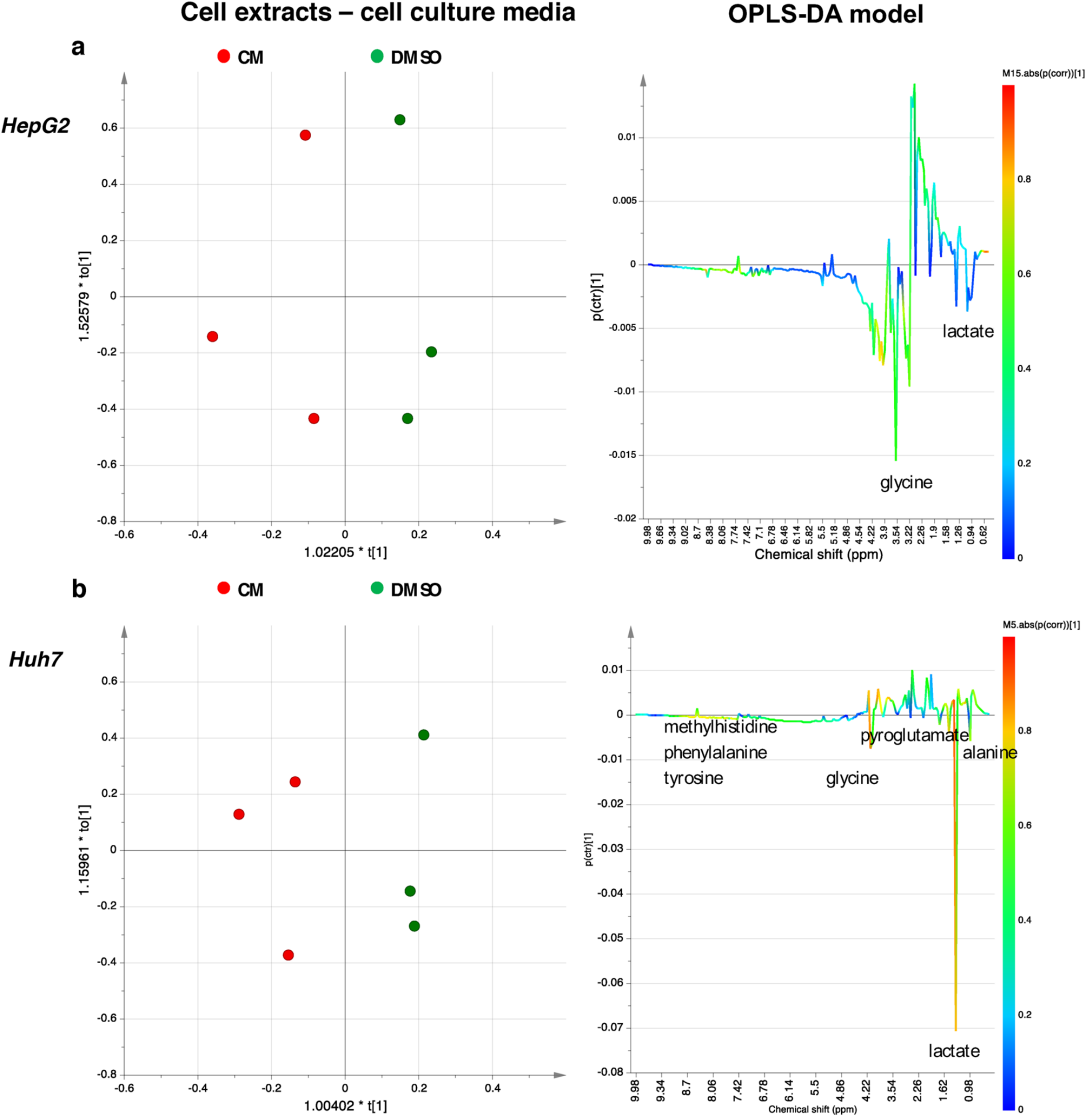
OPLS-DA scores plots (left panel) and corresponding coefficient loading plots (right panel) derived from the ^1^H-NMR spectra of HepG2 (**a**) and Huh7 (**b**) cell culture media samples obtained from different groups. *DMSO* Vehicle treatment, *CM* *Crithmum maritimum* treatment.

**Figure 6:**
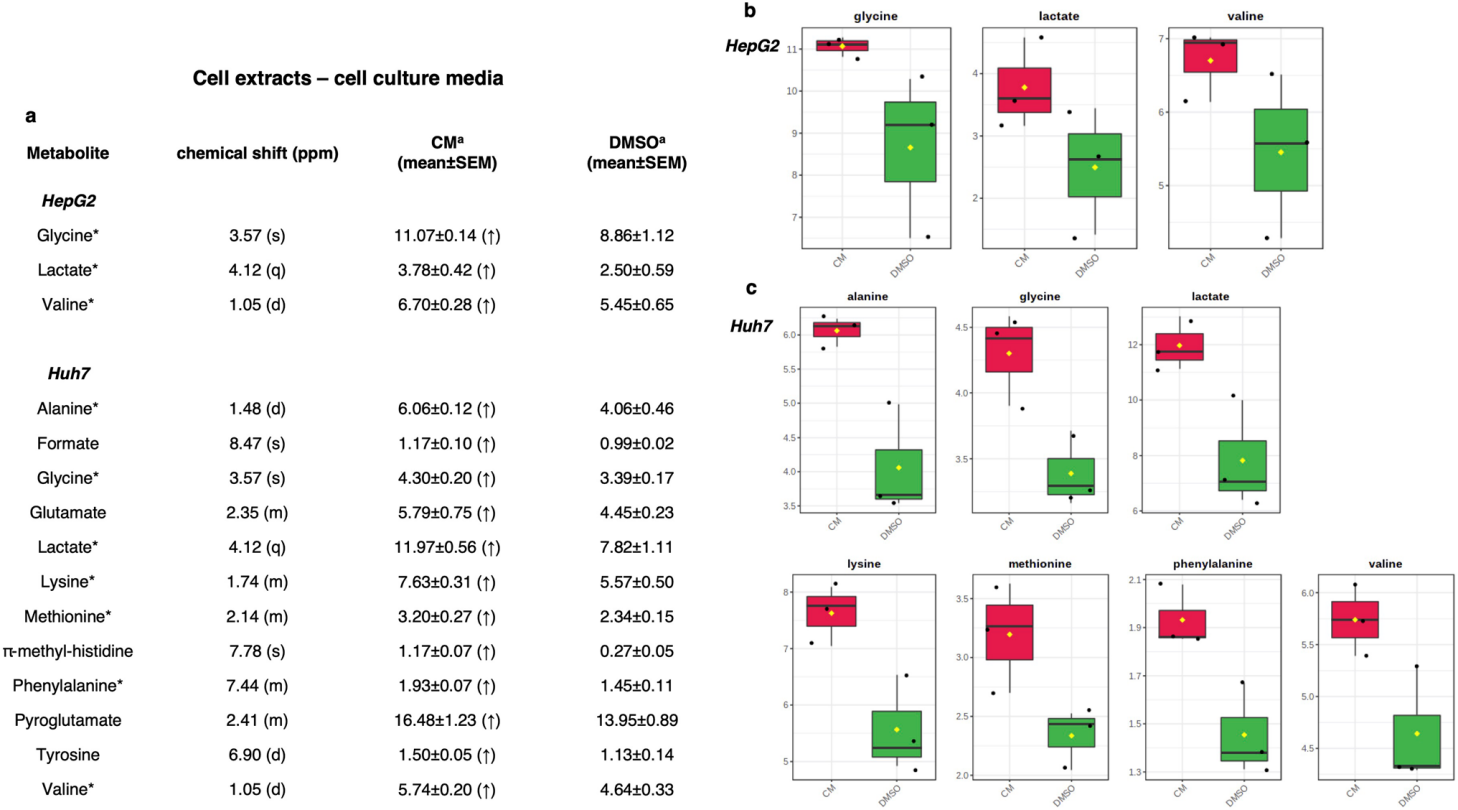
(**a**) Quantitative comparison of metabolites found in cell culture media. Mean and relative standard error mean refers to the relative integrals of metabolites, determined from cell culture media 1D ^1^H-NMR spectra of each group (CM and DMSO treatments). Letters in parentheses indicate peak multiplicities (s, singlet; d, doublet, dd, doublet of doublet; q, quartet, m, multiplet). * A *p*-value threshold of 0.05 was obtained from t-test analysis. (**b**,**c**) Box-and-Whisker plots illustrating the significant metabolites trend grouped according to *CM* *Crithmum maritimum* treatment, *DMSO* Vehicle treatment, derived from the ^1^H-NMR spectra of (**a**) HepG2 and (**b**) Huh7 cell culture media.

For both cell extracts (aqueous and lipid fractions) and cell culture media, 1D and 2D COSY, HSQC, HMBC, and J-*resolved* NMR spectra were used to accurately identify and assign metabolites according to literature data^28–32^. Then, a multivariate analysis (MVA) was performed. In a first step, a general overview for both the HepG2 and Huh7 cell lines was obtained by unsupervised PCA (Principal Components Analysis) analyses, while supervised OPLS-DA models were used to elucidate the most reliable class-discriminating variables that were highly diagnostic for CM and DMSO group separation. OPLS-DA models (both obtained with one predictive and one orthogonal component, R^2^X = 0.71, R^2^Y = 0.89, Q^2^ = 0.61, and R^2^X = 0.43, R^2^Y = 0.99, Q^2^ = 0.66), clearly evidenced differences in the concentrations of polar metabolites of the two cell lines for the treated compared with untreated samples (Fig. 3). In particular, by examining the color-coded coefficient loadings (S-line plots) for the OPLS-DA model reported in Fig. 3a, a decrease in levels of alanine (1.5 ppm), choline/phosphocholine (3.22 ppm), glycerol (3.66 ppm), glycine (3.56 ppm), lactate (1.33 ppm) and lysine (1.74 ppm) was observed for extracts of HepG2 cells treated with CM compared with vehicle (DMSO). On the other hand, relative low levels of acetate (1.92 ppm), methionine (2.14 ppm), choline (3.20 ppm), lactate (1.34 ppm), lysine (1.74 ppm), taurine (3.42 ppm), glycine (3.56 ppm) and glutamate (2.38 ppm) levels were observed in Huh7 extracts treated with CM compared to vehicle (DMSO). Moreover, the discriminant metabolites between CM treated and vehicle (DMSO) for both HepG2 and Huh7 cells were evaluated by the integration of the corresponding unbiased selected NMR signals. The t-test analysis was performed on the normalized peak integral areas of metabolites discriminating the two groups, with levels of statistical significance at least at p-values < 0.05 with a 95% confidence level (see Supplementary Table S1 online**)**. For metabolites defined in the ^1^H-NMR spectra by multiple patterns of signals, the non-overlapping signals were chosen, and relevant data obtained were expressed as normalised integrals for the discriminating metabolites (Figs. 4a**–**c, 5 and 6).

### ***Crithmum maritimum*** ethyl acetate extract modifies metabolite profile in cell culture media as evaluated by ^1^H-NMR spectroscopy

**Figure 7:**
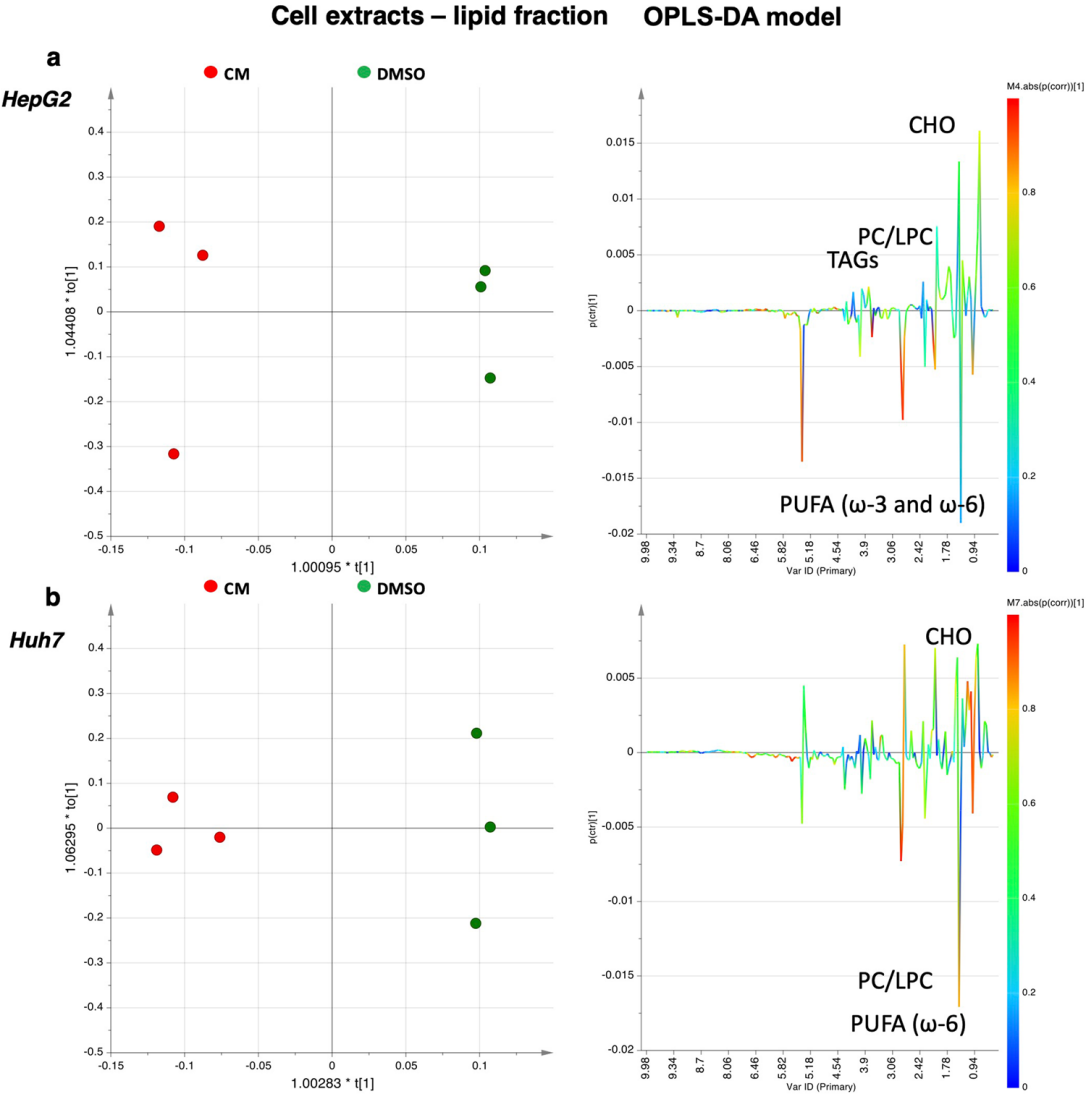
OPLS-DA scores plots (left panel) and corresponding coefficient loading plots (right panel) derived from the ^1^H-NMR spectra of (**a**) HepG2 and (**b**) Huh7 cell lipid extracts obtained from different groups. *DMSO* Vehicle treatment, *CM* *Crithmum maritimum* treatment.

**Figure 8:**
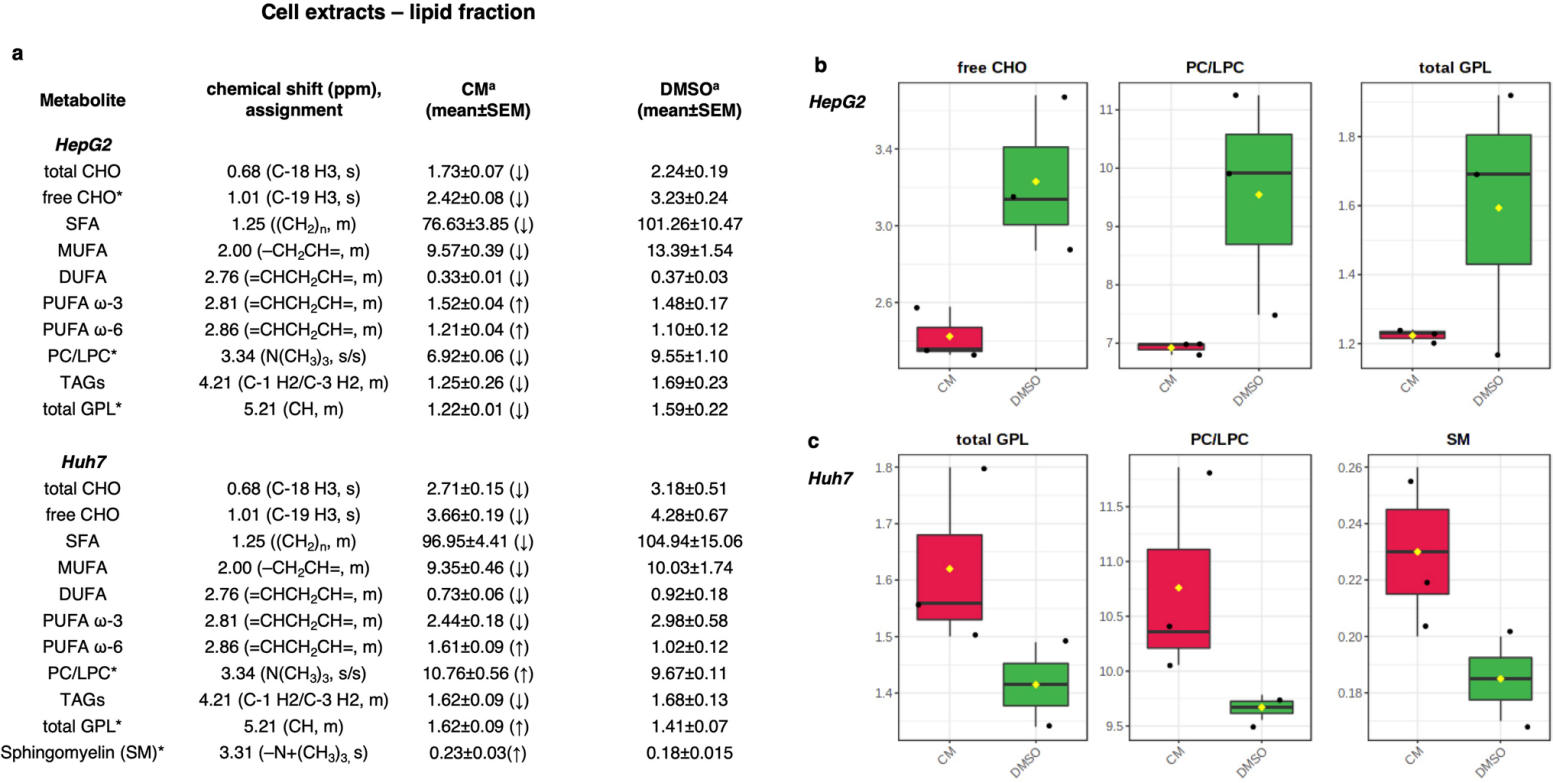
(**a**) Quantitative comparison of metabolites found in cell lipid extracts. Mean and relative standard error mean refers to the relative integrals of metabolites, determined from lipid cell extract 1D ^1^H-NMR spectra of each group (CM and DMSO treatments). Letters in parentheses indicate the peak multiplicities (*s* singlet, *d* doublet, *dd* doublet of doublet, *q* quartet, *m* multiplet). *A *p*-value threshold of 0.05 was obtained from t-test analysis. (**b**,**c**) Box-and-Whisker plots illustrating the significant metabolites trend grouped according to *CM* *Crithmum maritimum* treatment, *DMSO* DMSO treatment**,** derived from the ^1^H-NMR spectra of (**a**) HepG2 and (**b**) Huh7 cell lipid extracts.

Similarly, the corresponding OPLS-DA models were obtained for cell culture media samples (Fig. 5a,b). Both models were obtained with one predictive and one orthogonal component, R^2^X = 0.89, R^2^Y = 0.80, Q^2^ = 0.25 and R^2^X = 0.78, R^2^Y = 0.94, Q^2^ = 0.65. Data evidence a specific trend in the level of metabolites in *Crithmum maritimum*-treated samples when compared with the vehicle (DMSO). In HepG2 cell culture media only glycine, lactate, and valine were increased in *Crithmum maritimum*-treated samples. On the other hand, in Huh7 samples, relatively higher levels of alanine, formate, glycine, glutamate, lactate, lysine, methionine, methylhistidine, pyroglutamate, phenylalanine, tyrosine, and valine were detected. Relevant metabolites found by MVA were evaluated by the integration of the unbiased signal in the ^1^H-NMR spectra, and t-test analysis was used to determine statistical significance. Results, expressed as normalized integrals for the discriminating metabolites, are reported in Figs. 6a–c, 7 and 8.

### ***Crithmum maritimum*** ethyl acetate extract modifies metabolite profile in lipid cell extracts as evaluated by ^1^H-NMR spectroscopy

Finally, we analysed the lipid fraction in *Crithmum maritimum*-treated HepG2 and Huh7 cells compared with vehicle (DMSO) samples. Non-polar extracts were characterized by the presence of esterified lipids (TAGs, PUFA, DUFA, MUFA, SFA) and minor components, such as sterols (i.e., cholesterol, 0.68 ppm for total cholesterol and 1.01 ppm for free cholesterol) and phospholipids (partially overlapping singlets at 3.34 due to the N(CH_3_)_3_ groups of phosphatidylcholine (PC) and lysophosphatidylcholine (LPC). As shown by the OPLS-DA models (Fig. 7), both obtained with one predictive and one orthogonal component, R^2^X = 0.77, R^2^Y = 0.99, Q^2^ = 0.84 and R^2^X = 0.66, R^2^Y = 0.98, Q^2^ = 0.71, a trend can be detected in both HepG2 and Huh7 samples treated with *Crithmum maritimum* when compared to vehicle (DMSO). A moderate relative higher content of fatty acids (particularly PUFA ω-3 and ω-6, and MUFA) was observed in *Crithmum maritimum*-treated extracts of HepG2 cells if compared to vehicle (DMSO). Indeed, relatively lower levels of cholesterol (both free and total), SFA, DUFA, TAGs, and phospholipids (PC, LPC, GPL) were detected in *Crithmum maritimum*-treated HepG2 cells. A similar trend was observed in Huh7 cells, except for PUFA ω-3. Relevant metabolites found by MVA were evaluated by the integration of the unbiased signal in the ^1^H-NMR spectra, and the t-test accounted for the significance of variation. Results, expressed as normalized integrals for the discriminating metabolites, are reported in Fig. 8a–c.

## Discussion

Studying the effects of plants for the prevention and cure of different diseases is a growing area of interest for the scientific community^33^. Several plant-derived compounds are under evaluation as potential anti-cancer drugs^34^, and some are being tested in clinical trials^35^. Scientific evidence supports the view that beneficial effect on health lies in the complex blend of compounds characteristic of each plant, rather than to the action of a single or few molecules^36,37^. Indeed, the anticancer effect of a mixture of compounds from desert plant extracts has been described in HCC cells^38^. Also, some reports demonstrated the effectiveness of the ethyl acetate extract of three traditional plants on cervical cancer^39^ and HCC^40,41^.

We have previously shown that *Crithmum maritimum* ethyl acetate extract exerts an evident cytostatic effect in HCC cell lines^9^. In this study, we extended the analysis using a metabolomic approach to obtain additional insights for a better understanding of the mechanism of action of this plant extract. Our results clearly show that the metabolite signature notably changed in both cell lines upon treatment with the *Crithmum maritimum* ethyl acetate extract. This offers an interesting framework, which outlines a multi-target effect on key metabolites (Fig. 1c) that are altered in HCC. Regarding metabolites found in the aqueous fraction, lactate is well-known to be increased in tumours, as a consequence of the Warburg effect^42^. We observed a significant decrease in intracellular lactate production in both cell lines, which was mirrored by a considerable increase in cell culture media. We also found a substantial decrease in several amino acids, such as valine, tyrosine, phenylalanine, leucine, isoleucine, and glycine in both HCC cell lines. Indeed, it was reported that the level of these amino acids was increased in sera from HCC patients compared to healthy subjects^43^. This trend was confirmed in another metabonomic study which compared HCC tissues and contiguous healthy liver tissue^44^. The same study also reported a significant increase of lactate, glycerol, choline, and phosphocholine, and key amino acids, namely leucine, valine, alanine, glutamate, and glutamine in HCC tissues^44^. The increased level of choline and phosphocholine, described in HCC in vivo by magnetic resonance spectroscopy (MRS)^45,46^, can be explained by the increased demand for membrane phospholipids due to the accelerated proliferative rate in cancer cells.

As for the lipid fraction, upon treatment with *Crithmum maritimum* ethyl acetate extract, we found an important decrease of saturated fatty acids (SFAs), triglycerides (TAGs), total cholesterol (total CHO), and mono- and di-unsaturated fatty acids (MUFA and DUFA). Moreover, we observed a significant decrease in free cholesterol and an increase in ω-6 PUFA in both cell lines. ω-3 PUFA were increased in HepG2 cells and slightly decreased instead in Huh7. Recent studies have underlined the role of lipid homeostasis for tumour development and progression. In a study performed in 20 patients, the level of PUFA was significantly lower compared to healthy liver tissue^47^. More recently, cholesterol was also found to be essential for cancer cell metabolism and was proposed as a promising therapeutic target^48^. We also observed a slight difference in terms of response to the *Crithmum maritimum* ethyl acetate extract between the two employed cell lines, which may be due to their different genetic background which accounts for HCC heterogeneity^49^. Indeed, our study shows that the multi-target action of *Crithmum maritimum* ethyl acetate extract is effective upon this heterogeneity. This supports the idea of employing a blend of plant-derived bioactive compounds against cancer. This view is further corroborated by the observation that the effect of *Crithmum maritimum* is still maintained upon extracellular acidic pH conditions. Moreover, since we observed that *Crithmum maritimum* is also effective in a non-HCC cell line, further investigations should be extended to other malignancies.

Overall, our data demonstrate that *Crithmum maritimum* ethyl acetate extract normalise the metabolic profile of HCC cells by modulating key metabolic processes. Such pathways were reported to contribute to the tumorigenic phenotype of HCC. As depicted in Fig. 1c, this effect is exerted through a multi-target synergistic effect, which acts on several key "leverage points" of HCC cells, such as glucose metabolism by counteracting the Warburg effect, protein anabolism by decreasing amino acid level, and membrane biosynthesis by reducing choline and phosphocholine. Lipid homeostasis was also affected by lowering the levels of cholesterol, MUFA, and DUFA, and by increasing PUFA. Taken together, these effects provide a biochemical basis for the observed cytostatic effect of *Crithmum maritimum* in HCC cells.

Our findings identify a metabolic basis for *Crithmum maritimum*-mediated cytostasis and pave the way for further investigations on the multiple mechanisms by which this plant exerts such effect. This is in line with the need of developing systems pharmacology approaches for improving the treatment of tumours. Plant secondary metabolites have evolved for defence purposes against different categories of aggressors. For this reason, these metabolites are thought to interact with a plethora of targets in biological systems. The synergistic action of many phytochemicals may provide effective tools in disease management, including cancer^50,51^. In the case of *Crithmum maritimum*, the synergy of several secondary metabolites, such as phytosterols, glycerolipids, polyacetylenes, hydroxycinnamic acids, and hydroxybenzoic acid derivatives accounts for the observed cytostatic effect in HCC. A systems pharmacology approach using *Crithmum maritimum* will offer concrete opportunities for developing new effective therapeutic opportunities for HCC by integrating established drug protocols with plant-derived formulations.

## Materials and methods

### Chemicals

All of the solvents employed for the extraction procedure and analytical determinations were of analytical grade and HPLC grade. Hexane was purchased from Honeywell, ethyl acetate, methanol, and ethanol from Sigma-Aldrich. Dimethyl sulfoxide (DMSO) was purchased from Corning [cat. # 25-950-CQC].

All chemical reagents used for ^1^H-NMR analyses were of analytical grade. CDCl_3_, CD_3_OD (99.8 atom%D), TMS (0.03% v/v) were purchased from Armar Chemicals (Döttingen, Switzerland).

### Plant material and extraction procedure

*Crithmum maritimum* L. was harvested on the Apulian coast in the Bari metropolitan area (Bari S. Spirito). The extraction procedure was performed as previously described^9^. Briefly, after the whole plant was desiccated at 60 °C, the powder was obtained by mechanical trituration. The average yield of extraction obtained was ≈ 96% fresh/dried plant material. The powder was then subjected to the first extraction with hexane, by adding 150 mL of hexane to 150 mg plant powder in a conical flask and leaving the mixture under gentle orbital shaking for 48 h. Then, after filtration, the extract was let to evaporate at room temperature under a chemical hood. The extraction protocol described above was then repeated for ethyl acetate, methanol, and ethanol. Obtained extracts were then solubilised in cell culture-grade DMSO. The concentration of the extract was determined by high-pressure liquid chromatography (HPLC) as previously described^9^.

### Cell culturing

Huh7 and HepG2 cell lines were purchased from JCRB cell bank [cat. # JCRB0403 and cat. # JCRB1054, respectively]. HeLa cell line was purchased from ATCC cell bank [cat. # ATCC CCL-2). All of the three cell lines were grown in Dulbecco's modified Eagle's medium (DMEM) with 1 g/L glucose, 4 mM glutamine, 1 mM sodium pyruvate [Corning cat. #10-014-CVR], supplemented with 1X MEM-Nonessential Amino Acids [Corning cat. # 25-025-CIR], 20 mM Hepes Buffer [Aurogene cat. # AU-L0180-500], 1X Antibiotic–Antimycotic solution [Corning cat. # 30-004-CI] and 10% Foetal Bovine Serum (FBS) [Corning cat. # 35-079-CV]. Cells were grown under standard culturing conditions (humidified atmosphere, 37 °C, and 5% CO_2_).

For ^1^H-NMR experiments, cells were treated with *Crithmum maritimum* ethyl acetate extract 0.5 μM for 48 h. Then, 1 mL of cell culture media was harvested from vehicle- and *Crithmum maritimum*-treated cells. Cell pellets were obtained after centrifugation of scraped and harvested cells. For cell proliferation experiments, cells were treated with the four different *Crithmum maritimum* extracts at 0.5 μM for 72 h and then processed for crystal violet staining as described below in this section. Cell proliferation experiments in acidic pH conditions were performed using DMEM medium acidified at pH 6.8 with 1 M HCl as previously reported^52^. Cells were plated in the acidified DMEM, treated with the four different *Crithmum maritimum* extracts at 0.5 μM for 72 h, and then processed for crystal violet staining.

### Sample preparation for ^1^H-NMR analysis

Cell samples: NMR samples were obtained from Huh7 and HepG2 cell pellets and cell culture medium (CCM). Cell extracts were prepared according to a modified Bligh and Dyer two-step method^53–55^ with a methanol/chloroform/water mixture, which was further treated to separate polar and lipophilic fractions. The hydrophilic and lipophilic phases were separated and dried using a SpeedVac concentrator. Lipid fractions were dissolved in 600 µL CDCl_3_ and transferred to a 5-mm NMR tube. Polar fractions and CCM samples were resuspended in a D_2_O phosphate buffer (0.1 M K_2_HPO_4_, 2 mM sodium azide, pH 7.4)^29,55^.

For *Crithmum m.* extract: to obtain a satisfactory dissolution of polar and non-polar analytes, the ethyl acetate extract was resuspended in a 1:1 CD_3_OD:CDCl_3_ (containing tetramethylsilane, TMS 0.03 v/v %) mixture. 600 μL of the mixture was transferred into a 5-mm NMR tube and immediately subjected to NMR analysis.

### ^1^H-NMR measurements

#### Cell samples

For each aqueous sample a 1D sequence with pre-saturation and composite pulse for selection (zgcppr Bruker standard pulse sequence) was acquired, with 256 transients, 16 dummy scans, 5 s relaxation delay, size of fid of 64 K data points, a spectral width of 12,019.230 Hz (20.0276 ppm) and an acquisition time of 2.73 s, and with chemical shift referencing to trimethylsilyl propionic-2,2,3,3-d 4 acid sodium salt (TSP) signal (δ = 0.00 ppm). The resulting FIDs were multiplied by an exponential weighting function corresponding to a line broadening of 0.3 Hz before Fourier transformation, automated phasing, and baseline correction. For each lipid extract a one-dimensional experiment (zg Bruker pulse program) was run with 128 scans, 64 K time domain, spectral width 20.0276 ppm (12,019.230 Hz), 5 s delay, p1 8 µs and 2.73 s acquisition time. All spectra were referenced to the tetramethylsilane (TMS) signal (δ = 0.00 ppm). Metabolites were assigned based on 2D NMR spectra analysis (2D ^1^H J*res*, ^1^H COSY, ^1^H-^13^C HSQC, and HMBC) and by comparison with published data^29–31,56,57^.

*Crithmum maritimum* ethyl acetate extract: Sample characterisation was performed by acquiring a ^1^H-NMR spectrum with water signal suppression (Bruker pulseprogram zgcppr), in a spectral window of 20.0276 ppm (12,019.230 Hz), 128 scans, and a 90° pulse of 7.55 µs, 64 K time domain, spectral width 20.0276 ppm (12,019.230 Hz), 5 s delay, and 2.73 s acquisition time. After the acquisition, the standard FID processing procedures were carried out, by using TopSpin 3.5 (Bruker, Biospin, Italy), such as the Fourier transform (a mathematical operation that converts signals into a frequency spectrum), the phase and baseline correction, and 0.3 Hz line broadening. All the ^1^H-NMR spectra were calibrated to the internal standard TMS (δ = 0.00 ppm). Characterization of metabolites was performed by the analysis of two-dimensional homo- and heteronuclear NMR spectra (2D ^1^H J-resolved, ^1^H COSY, ^1^H-^13^C HSQC, and HMBC) and by comparison with published data^17,21,22,24–27,58^.

All measurements were performed on a Bruker Avance III 600 Ascend NMR spectrometer (Bruker, Bruker, Ettlingen, Germany) operating at 600.13 MHz for ^1^H observation, equipped with a z-axis gradient coil and automatic tuning-matching (ATM). Experiments were acquired at 300 K in automation mode after loading individual samples on a Bruker Automatic Sample Changer, interfaced with the software IconNMR (Bruker).

### Multivariate data analysis

To account for variations of the overall concentrations of samples, NMR spectra were processed using Topspin 3.6.1 and Amix 3.9.13 software (Bruker, Biospin, Italy) for simultaneous visual inspection and the successive bucketing process. NMR spectra were segmented in rectangular buckets of fixed 0.04 ppm width and integrated. For aqueous cell fractions, spectral regions between 5.00–4.50 ppm were discarded because of the residual peak of water signal, while for CCM NMR spectra, signals of Hepes and DMSO and its affected neighbouring regions between 3.87–3.77 and 3.20–2.55 ppm were discarded. For lipid extract^, 1^H-NMR spectra—regions between 7.75–6.75, 3.48–3.25, and 1.70–1.44 ppm—were excluded before analysis due to the residual peaks of solvents (chloroform, methanol, and residual water signals). For each of the resulting data sets, a matrix was obtained, made of the bucketed ^1^H-NMR spectra values (columns) measured for each sample (rows). The mean centred and Pareto scaling procedures were applied to the data before multivariate statistical analyses, to attenuate the effect of dominant variables and noise while amplifying weak signals to the largest possible^32,59,60^.

### Quantitative analysis of metabolites performed by ^1^H-NMR spectroscopy

By ^1^H-NMR spectroscopy, metabolites of interest were quantified by analysing the integrals of selected distinctive unbiased NMR signals. Integral values for metabolite quantitative analysis were referred to as the internal standard (TSP and TMS for aqueous and lipid extracts, respectively). Results, represented as mean intensities and standard error mean of the selected NMR signals, were validated by the univariate t-test^61–63^. Levels of statistical significance were at least at p-values < 0.05 with a 95% confidence level. The univariate analysis made on the integral area of metabolites between groups was performed using MeTPA MetaboAnalyst software^64^.

### Cell proliferation assays

End-point proliferation was assessed by crystal violet staining 72 h after plant extract addition. Crystal violet [Sigma-Aldrich cat. #C3886] was diluted in EtOH/H_2_O 10% v/v to obtain a 1 mg/mL solution. Before Crystal violet addition, cells were fixed in 4% paraformaldehyde. Colour elution was performed with 10% acetic acid and absorbance was measured at λ = 595 nm using an iMark plate reader [Bio-Rad cat. #168–1135].

### Statistical analyses

Concerning ^1^H-NMR, relevant metabolites detected by MVA were evaluated by the integration of the unbiased signal in the ^1^H-NMR spectra, and statistical significance, set at p < 0.05, was assessed by t-test.

Cell proliferation data were analysed by Two-way ANOVA, followed by Dunnett’s multiple comparisons test. For data not normally distributed, the Kruskal–Wallis test followed by Dunn’s multiple comparisons test was employed.

## Supplementary Information

Supplementary Information
