# Supplementary material for: 1H-NMR metabolomics reveals a multitarget action of Crithmum maritimum ethyl acetate extract in inhibiting hepatocellular carcinoma cell growth
Source: Sci Rep. 2021 Jan 13;11:1259. doi: 10.1038/s41598-020-78867-1 (PMC7806899; doi:10.1038/s41598-020-78867-1)
Supplement: Supplementary file 1 — Supplementary Information [file 41598_2020_78867_MOESM1_ESM.docx]

**Supplementary Information for:**

**^1^H-NMR metabolomics reveals a multitarget action of *Crithmum maritimum* ethyl acetate extract in inhibiting hepatocellular carcinoma cell growth**

**Davide Gnocchi^1^, Laura Del Coco^2^, Chiara Roberta Girelli^2^, Francesca Castellaneta^1^, Gianluigi Cesari^3^, Carlo Sabbà^1^, Francesco Paolo Fanizzi^2,*^, Antonio Mazzocca^1,*^**

^1^ Interdisciplinary Department of Medicine, University of Bari School of Medicine, Piazza G. Cesare, 11 - 70124 Bari, Italy

^2^ Department of Biologic and Environmental Sciences and Technologies, University of Salento, 73100 Lecce, Italy.

^3^ CIHEAM - Mediterranean Agronomic Institute of Bari, Department of Organic Agriculture, 70010 Valenzano (BA), Italy

^*^ Corresponding authors:

Antonio Mazzocca, M.D., Ph.D. Professor of Pathology & Laboratory Medicine, Interdisciplinary Department of Medicine, University of Bari School of Medicine, Piazza G. Cesare, 11 I-70124 Bari, Italy

Tel.: +39 080 5593593
E-mail address: [antonio.mazzocca@uniba.it](mailto:antonio.mazzocca@uniba.it)

Francesco Paolo Fanizzi, Department of Biologic and Environmental Sciences and Technologies, University of Salento, 73100 Lecce, Italy

E-mail address: [fp.fanizzi@unisalento.it](mailto:fp.fanizzi@unisalento.it)

**Supplementary Figure S1.** Effect of the different *Crithmum maritimum* extracts on cell proliferation in the HeLa cell line as determined by crystal violet staining. Cells were treated for 72 h with the four different *Crithmum maritimum* extracts at 0.5 μM. **** p< 0.0001, as determined by the Kruskal-Wallis test followed by Dunn’s multiple comparisons test. n.s. not significative. Results are expressed as the mean ± s.e.m. of at least three independent biological replicates, each conducted in triplicate.

**Supplementary Figure S2.** Effect of the different *Crithmum maritimum* extracts on cell proliferation in two different HCC cell lines (Huh7 and HepG2) under acidic extracellular pH conditions as determined by crystal violet staining. Cells were treated for 72 h with the four different *Crithmum maritimum* extracts at 0.5 μM. **** p< 0.0001, *** p< 0.001, ** p< 0.01 as determined by 2-way ANOVA analysis followed by Dunnett’s multiple comparisons test. n.s. not significative. Results are expressed as the mean ± s.e.m. of at least three independent biological replicates, each conducted in triplicate.

**Cell extracts - aqueous fractions**

| Metabolite | chemical shift (ppm) | t.stat | p.value | -log_10_(p) | FDR |
| --- | --- | --- | --- | --- | --- |
| ***HepG2*** |  |  |  |  |  |
| Alanine | 1.48 (d) | -0.51 | 0.64 | 0.20 | 0.68 |
| Choline***** | 3.20 (s) | -3.67 | 0.02 | 1.67 | 0.36 |
| PC***** | 3.22 (s) | -2.44 | 0.07 | 1.15 | 0.50 |
| GPC | 3.23 (s) | -2.14 | 0.10 | 1.01 | 0.50 |
| Glycerol***** | 3.66 (m) | -1.99 | 0.12 | 0.93 | 0.50 |
| Glycine | 3.56 (s) | -1.10 | 0.33 | 0.48 | 0.58 |
| Lactate | 1.33 (d) | -1.01 | 0.37 | 0.43 | 0.58 |
| Lysine | 1.74 (m) | -0.81 | 0.46 | 0.34 | 0.58 |
| ***Huh7*** |  |  |  |  |  |
| Acetate | 1.92 (s) | -1.13 | 0.32 | 0.49 | 0.60 |
| Choline | 3.20 (s) | -0.91 | 0.41 | 0.38 | 0.67 |
| PC | 3.22 (s) | 1.15 | 0.31 | 0.50 | 0.60 |
| GPC | 3.23 (s) | -0.25 | 0.81 | 0.09 | 0.95 |
| DMA | 2.74 (s) | -0.61 | 0.57 | 0.24 | 0.75 |
| Glycine***** | 3.56 (s) | -3.81 | 0.02 | 1.72 | 0.22 |
| Glutamate***** | 2.38 (m) | -3.02 | 0.04 | 1.41 | 0.22 |
| Lactate | 1.33 (d) | -1.44 | 0.22 | 0.65 | 0.58 |
| Lysine | 1.74 (m) | -1.68 | 0.17 | 0.78 | 0.54 |
| Methionine | 2.14 (m) | -0.65 | 0.55 | 0.26 | 0.75 |
| Pyruvate | 2.40 (s) | 0.16 | 0.88 | 0.06 | 0.95 |
| Taurine***** | 3.42 (dd) | -2.78 | 0.05 | 1.30 | 0.22 |

**Supplementary Table S1.** Table reporting statistical analysis of OPLS-DA model data shown in Figure 3. * A *p*-value threshold of 0.05 was obtained from t-test analysis.

**Cell culture media**

| Metabolite | chemical shift (ppm) | t.stat | p.value | -log_10_(p) | FDR |
| --- | --- | --- | --- | --- | --- |
| ***HepG2*** |  |  |  |  |  |
| Glycine* | 3.57 (s) | 2.13 | 0.10 | 1.00 | 0.56 |
| Lactate* | 4.12 (q) | 1.78 | 0.15 | 0.82 | 0.56 |
| Valine* | 1.05 (d) | 1.77 | 0.15 | 0.82 | 0.56 |
| ***Huh7*** |  |  |  |  |  |
| Alanine* | 1.48 (d) | 4.19 | 0.01 | 1.86 | 0.08 |
| Formate | 8.47 (s) | 1.86 | 0.14 | 0.87 | 0.19 |
| Glycine* | 3.57 (s) | 3.47 | 0.03 | 1.59 | 0.08 |
| Glutamate | 2.35 (m) | 1.70 | 0.17 | 0.78 | 0.21 |
| Lactate* | 4.12 (q) | 3.34 | 0.03 | 1.54 | 0.08 |
| Lysine* | 1.74 (m) | 3.53 | 0.02 | 1.61 | 0.08 |
| Methionine* | 2.14 (m) | 2.79 | 0.05 | 1.31 | 0.10 |
| π-methyl-histidine | 7.78 (s) | 2.42 | 0.07 | 1.14 | 0.12 |
| Phenylalanine* | 7.44 (m) | 3.59 | 0.02 | 1.64 | 0.08 |
| Pyroglutamate | 2.41 (m) | 1.66 | 0.17 | 0.77 | 0.21 |
| Tyrosine | 6.90 (d) | 2.48 | 0.07 | 1.16 | 0.12 |
| Valine* | 1.05 (d) | 2.83 | 0.05 | 1.33 | 0.10 |

**Supplementary Table S2.** Table reporting statistical analysis of OPLS-DA model data shown in Figure 5. * A *p*-value threshold of 0.05 was obtained from t-test analysis.

**Cell extracts – lipid fraction**

| Metabolite | chemical shift (ppm) | t.stat | p.value | -log_10_(p) | FDR |
| --- | --- | --- | --- | --- | --- |
| ***HepG2*** |  |  |  |  |  |
| total CHO | 0.68 (C-18 H3, s) | -2.51 | 0.07 | 1.18 | 0.18 |
| free CHO* | 1.01 (C-19 H3, s) | -3.22 | 0.03 | 1.49 | 0.18 |
| SFA | 1.25 ((CH_2_)_n_, m) | -2.21 | 0.09 | 1.04 | 0.18 |
| MUFA | 2.00 (–CH_2_CH=, m) | -2.41 | 0.07 | 1.13 | 0.18 |
| DUFA | 2.76 (=CHCH_2_CH=, m) | -0.98 | 0.38 | 0.42 | 0.48 |
| PUFA ω-3 | 2.81 (=CHCH_2_CH=, m) | 0.21 | 0.84 | 0.07 | 0.84 |
| PUFA ω-6 | 2.86 (=CHCH_2_CH=, m) | 0.87 | 0.44 | 0.36 | 0.48 |
| PC/LPC* | 3.34 (N(CH_3_)_3_, s/s) | -2.37 | 0.08 | 1.12 | 0.18 |
| TAGs | 4.21 (C-1 H2/C-3 H2, m) | -1.26 | 0.28 | 0.56 | 0.41 |
| total GPL* | 5.21 (CH, m) | -1.67 | 0.17 | 0.77 | 0.29 |
| ***Huh7*** |  |  |  |  |  |
| total CHO | 0.68 (C-18 H3, s) | 0.06 | 0.96 | 0.02 | 0.99 |
| free CHO | 1.01 (C-19 H3, s) | 0.06 | 0.96 | 0.02 | 0.99 |
| SFA | 1.25 ((CH_2_)_n_, m) | 0.97 | 0.40 | 0.39 | 0.75 |
| MUFA | 2.00 (–CH_2_CH=, m) | 1.04 | 0.38 | 0.42 | 0.75 |
| DUFA | 2.76 (=CHCH_2_CH=, m) | -0.26 | 0.81 | 0.09 | 0.99 |
| PUFA ω-3 | 2.81 (=CHCH_2_CH=, m) | -0.02 | 0.99 | 0.01 | 0.99 |
| PUFA ω-6 | 2.86 (=CHCH_2_CH=, m) | 5.97 | 0.01 | 2.03 | 0.12 |
| PC/LPC* | 3.34 (N(CH_3_)_3_, s/s) | 1.51 | 0.23 | 0.64 | 0.60 |
| TAGs | 4.21 (C-1 H2/C-3 H2, m) | 0.53 | 0.63 | 0.20 | 0.99 |
| total GPL* | 5.21 (CH, m) | 1.57 | 0.22 | 0.67 | 0.60 |
| Sphingomyelin (SM)* | 3.31 (–N+(CH_3_)_3,_ s) | 1.80 | 0.17 | 0.77 | 0.60 |

**Supplementary Table S3.** Table reporting statistical analysis of OPLS-DA model data shown in Figure 7. * A *p*-value threshold of 0.05 was obtained from t-test analysis.

**Supplementary Figure S3.** ^1^H-NMR typical spectrum (CD_3_OD: KH_2_PO_4_ buffer in D_2_O (pH 5.9), 1:1) of *Crithmum maritimum* powder. Diagnostic peaks of some metabolites are indicated in the expansions of the spectrum.

**Supplementary Figure S4.** ^1^H-NMR typical spectrum (CD_3_Cl_3_) of *Crithmum maritimum* hexane extract. Diagnostic peaks of some metabolites are indicated in the expansions of the spectrum.

**Supplementary Figure S5.** ^1^H-NMR typical spectrum (CD_3_OD) of *Crithmum maritimum* methanol extract. Diagnostic peaks of some metabolites are indicated in the expansions of the spectrum.

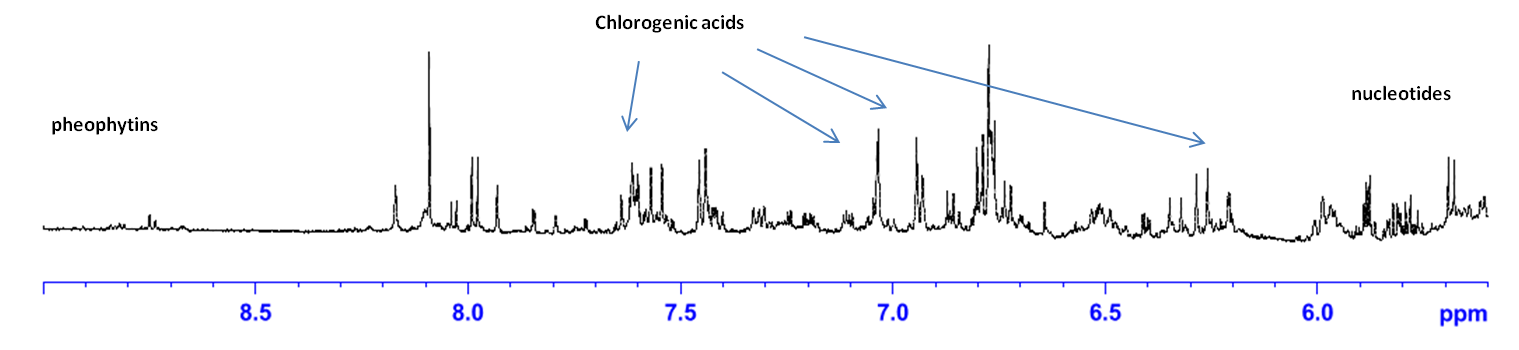


**Supplementary Figure S6.** ^1^H-NMR typical spectrum (CD_3_OD) of *Crithmum maritimum* ethanol extract. Diagnostic peaks of some metabolites are indicated in the expansions of the spectrum.
